# Macrophages-derived Factor XIII links coagulation to inflammation in COPD

**DOI:** 10.3389/fimmu.2023.1131292

**Published:** 2023-04-25

**Authors:** Erica Bazzan, Alvise Casara, Claudia Maria Radu, Mariaenrica Tinè, Davide Biondini, Eleonora Faccioli, Federica Pezzuto, Nicol Bernardinello, Maria Conti, Elisabetta Balestro, Fiorella Calabrese, Paolo Simioni, Federico Rea, Graziella Turato, Paolo Spagnolo, Manuel G. Cosio, Marina Saetta

**Affiliations:** ^1^ Department of Cardiac, Thoracic, Vascular Sciences and Public Health, University of Padova, Padova, Italy; ^2^ Department of Medicine, University of Padova, Padova, Italy; ^3^ Meakins-Christie Laboratories, Respiratory Division, McGill University, Montreal, QC, Canada

**Keywords:** COPD - chronic obstructive pulmonary disease, macrophage, coagulation, inflammation, injury

## Abstract

**Background:**

The local, extravascular, activation of the coagulation system in response to injury is a key factor mediating the resulting inflammatory response. Coagulation Factor XIIIA (FXIIIA) found in alveolar macrophages (AM) and dendritic cells (DC), by influencing fibrin stability, might be an inflammatory modifier in COPD.

**Aims:**

To study the expression of FXIIIA in AM and Langerin+DC (DC-1) and their relation to the inflammatory response and disease progression in COPD.

**Methods:**

In 47 surgical lungs, 36 from smokers (22 COPD and 14 no-COPD) and 11 from non-smokers we quantified by immunohistochemistry FXIIIA expression in AM and DC-1 along with numbers of CD8+Tcells and CXCR3 expression in lung parenchyma and airways. Lung function was measured prior to surgery.

**Results:**

The percentage of AM expressing FXIII (%FXIII+AM) was higher in COPD than no-COPD and non-smokers. DC-1 expressed FXIIIA and their numbers were higher in COPD than no-COPD and non-smokers. DC-1 positively correlated with %FXIII+AM (r=0.43; p<0.018). CD8+Tcells, which were higher in COPD than in no-COPD, were correlated with DC-1 (p<0.01) and %FXIII+AM. CXCR3+ cells were increased in COPD and correlated with %FXIII+AM (p<0.05). Both %FXIII+AM (r=-0.6; p=0.001) and DC-1 (r=-0.7; p=0.001) correlated inversely with FEV_1_.

**Conclusion:**

FXIIIA, an important link between the extravascular coagulation cascade and inflammatory response, is significantly expressed in alveolar macrophages and dendritic cells of smokers with COPD, suggesting that it could play an important role in the adaptive inflammatory reaction characteristic of the disease.

## 1 Introduction

Work over the past 20 years has highlighted critical roles for coagulation system components in a wide range of physiological and pathological responses to injury. It is now well understood that coagulation system activity and extravascular fibrin deposits associated with tissue injury are not simply a response to gain hemostasis, but rather are key functional factors in mediating the resulting inflammatory response (1). Thus, factors that influence fibrin stability would not only affect the coagulation process but would also act as inflammatory modifiers.

Fibrin stability is provided by the Factor XIII (FXIII), a thrombin-activated transglutaminase enzyme that catalyzes the formation of e-N-(g-glutamyl)-lysine isopeptide bonds in protein substrate (1–4). The intravascular, plasmatic FXIIIA2B2 (A2 subunit macrophage origin, B2 subunit hepatic origin) has an established function in hemostasis, where its primary substrate is fibrin. A deficiency in FXIIIA2 manifests as a severe bleeding diathesis, underscoring its importance in this pathway (5).

FXIII is also present in an extravascular cellular form as a homodimer of the FXIIIA-subunits, denoted FXIIIA. The FXIII is expressed in cells of bone marrow and mesenchymal lineage, notably megakaryocytes and platelets, which FXIIIA level accounts for about 3% of the total protein (5), macrophages and dendritic cells (6–8). It is now clear that, in addition to its role in hemostasis, FXIIIA functions in a variety of other systems, ranging from wound healing and angiogenesis to inflammation (9). The association of FXIIIA to inflammatory diseases has been reported in pediatric chronic bronchoalveolar inflammatory abnormalities (10), asthma, and a mice model of rheumatoid arthritis (11), where the elimination of FXIIIA expression significantly limited disease progression.

In view of the important plausible inflammatory function of FXIIIA, its known presence in macrophages, its described role upon dendritic cells mobility and function, we hypothesized that FXIIIA might be expressed in alveolar macrophages in COPD lungs, which would point to a possible role of FXIIIA in the development of the adaptive inflammatory reaction which is a fundamental pathogenetic mechanism in COPD. To investigate this possibility, we studied the presence of FXIIIA in lungs obtained at surgery of smokers with and without COPD and in non-smokers and related it to lung inflammation and pulmonary function.

## 2 Methods

### 2.1 Subjects characteristics

We recruited three groups of subjects undergoing lung resection for appropriate clinical indications: 22 smokers with COPD, 11 of whom with severe COPD [GOLD stage III-IV: FEV_1_ <50% pred (12)] and 11 with mild to moderate COPD [GOLD stage I-II: FEV_1_ >50% pred (12)], 14 smokers without COPD and 11 non-smokers. Patients underwent clinical evaluation comprising pulmonary function tests before surgery. COPD was defined as FEV_1_/FVC less than 70% after bronchodilator, according to the recent GOLD 2023 document (12). None of the patients had past or present history of asthma, and no subject had an exacerbation of the disease within 1 month of surgery. The study conformed to the Declaration of Helsinki and all patients provided informed written consent before surgery. All aspects of this study were approved by the local ethics committee (reference No.0006045).

### 2.2 Immunohistochemical and confocal analysis

Lung tissue preparation and immunohistochemistry were performed as previously described (13–16). Briefly, lungs were fixed in 4% formaldehyde and 5µm thick sections were cut and processed for morphometric and immunohistochemical analysis. To quantify FXIIIA expression in alveolar macrophages in, at least 20 non-consecutive high-power fields (hpf) and at least 100 macrophages were evaluated for each subject. Results were expressed as percentage of FXIIIA+ macrophages over the total number of macrophages examined. To quantify CD8+ T-lymphocytes and CXCR3+ cells in alveolar walls, at least 10 non-consecutive fields were evaluated, and results were expressed as number of CD8+ and CXCR3+ cells per mm of alveolar wall. CXCR3 was also quantified in alveolar macrophages and results were expressed as percentage of CXCR3+ macrophages over the total number of macrophages examined. Langherin+ dendritic cells, CD8 T-lymphocytes and CXCR3+ cells were identified and quantified in peripheral airways and results were expressed as number of positive cells per square millimeter (details in online supplement). Negative control for nonspecific binding were processed, either omitting the primary antibody or using isotype IgG, and revealed no signal. All analyses were performed with a Leica light microscope and a video recorder linked to a computerised image analysis system (Leica LAS w3.8).

In three cases from each group, confocal microscopy was performed to study the expression of FXIIIA in DC and possible co-expression of FXIIIA and CXCR3 in AM (details in the online supplement).

### 2.3 Statistical analysis

Cases were coded, and measurements were made without knowledge of clinical data. The patient’s characteristics were expressed using the mean ± SD for clinical data or median (range) for morphological data. For continuous variables, normal distributions were tested using the Shapiro-Wilk test. One way ANOVA with Turkey’s multiple comparison test were used to analyse the parametric clinical data. The non-parametric Kruskal-Wallis test and Dunn test for multiple comparison were used to evaluated the quantitative morphological data and the difference among groups. Correlation coefficients were calculated using the nonparametric Spearman rank method. The intraobserver measurements correlation coefficient for FXIIIA was 0.94, while the interobserver correlation coefficient was 0.90. The intraobserver measurements correlation coefficient for all other cell counts ranged from 0.90 to 0.94, while the interobserver correlation coefficient ranged from 0.88 to 0.90. All data were analysed by SPSS statistical software (version 3.5.2). P < 0.05 was considered statistically significant.

## 3 Results

### 3.1 Clinical characteristics

The clinical characteristics of the subjects in the study are shown in **Table 1**. Thirty-six were smokers, 22 of whom with COPD and 14 without COPD. Eleven had never smoked. There were no demographic differences among the groups and the smoking history was not different in smokers with and without COPD. Among the subjects with COPD, 11 had GOLD stage III-IV (severe disease) and 11 GOLD stage I-II (mild/moderate disease).

**Table 1:** Clinical characteristics of the subjects in the study cohort.

	COPD (n=22)	Smokers w/o COPD (n=14)	Non-Smokers (n=11)	p
Subjects examined (M/F)	18M/4F	13M/1F	5M/6F	
Age (years)	64 ± 8	64 ± 9	69 ± 5	N.S.
Smoking history (pack-year)	48 ± 21	39 ± 24	–	N.S.
FEV1 (%pred)	51± 20*	99± 10	105 ± 16	0.001
FEV1/FVC (%)	49 ± 16*	79 ± 6	80 ± 4	0.001
PaO2 (mmHg)	77 ± 15	84 ± 8	84 ± 8	N.S.
PaCO2 (mmHg)	40 ± 5	40± 10	38 ± 3	N.S.

Values are expressed as mean ± SD.

The statistical analysis was performed using One way ANOVA with Turkey as a post-hoc test.

*Significantly different from Smokers w/o COPD and non-smokers (p < 0.001). N.S., non significant.

### 3.2 Histological findings

#### 3.2.1 Factor XIIIA (FXIIIA) in lung alveolar macrophages

**Figure 1:**
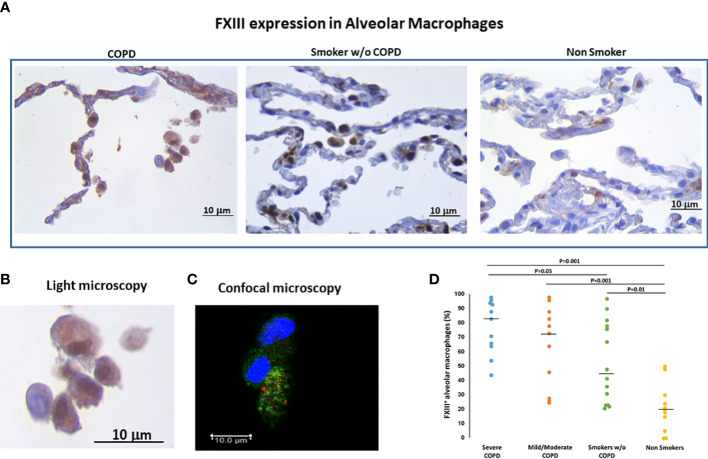
FXIIIA expression in alveolar macrophages (AM). **(A)** Immunohistochemistry of FXIIIA+AM in clusters of alveolar macrophages in smokers with and without COPD and non-smokers. FXIIIA immunoreactivity appears as a brown diffuse cytoplasmic granular pattern. **(B)** Enlargement of alveolar macrophages in COPD and **(C)** confocal macrophage images to show the cytoplasmic localization of FXIII: nuclei stained with DRAQ5 (blue), FXIIIA (green), CXCR3 (red). Scale bars= Bars: 10 μm. **(D)** Percentage of FXIIIA+AM over total AM in smokers with severe COPD (blue circles), mild/moderate COPD (orange circles), smokers without COPD (green circles) and non-smokers (yellow circles). Horizontal bars represent median values. Overall comparison by Kruskal-Wallis test (p<0.001) with Dunn’s multiple comparison test.

It was our aim to confirm that FXIIIA was expressed in alveolar macrophages (AM) and that there would be a differential expression among disease groups. The FXIIIA immunoreactivity was present in AM with a diffuse granular patterns at the cytoplasmic level as shown by light microscopy and confocal microscopy (**Figures 1A–C**). The percentage of AM expressing FXIIIA was significantly higher in smokers with COPD than in smokers without COPD [median(range) 75.5(27-98)% vs 45 (22-97)%; p=0.05] and non-smokers [median(range) 75.5(27-98)% vs 20(0-50)%; p=0.001], and was also higher in smokers without COPD than in non-smokers [median(range) 45(22-97)% vs 20(0-50)%; p=0.01; **Figure 1D**]. Within the COPD group, the percentage of AM expressing FXIIIA was similar in severe and in mild/moderate COPD. These results suggest that compared to individuals without COPD, alveolar macrophage FXIIIA expression is increased in COPD.

The expression of FXIIIA did not correlate with the amount of smoking in neither of the smoking groups and furthermore it was not different when active smokers and ex-smokers were compared [78(23-98)% vs 64(20-98)%], suggesting that the FXIII activation is mainly due to the disease.

#### 3.2.2 Dendritic cells in bronchiolar walls

**Figure 2:**
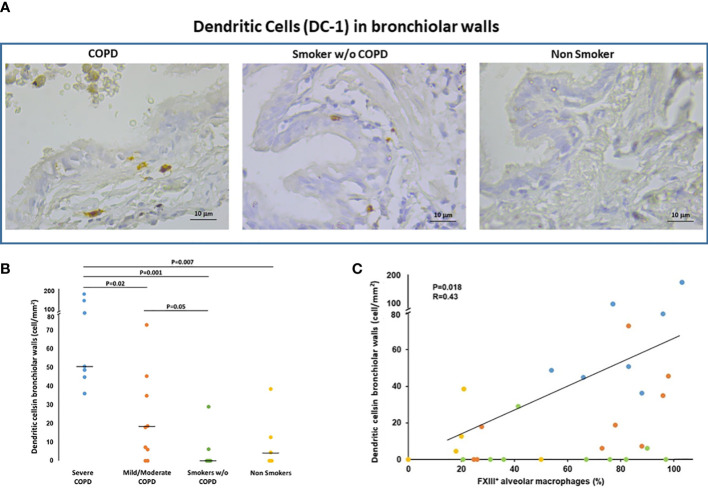
Dendritic cells (DC-1) in bronchiolar walls. **(A)** Immunohistochemistry of Langerin+ cells (DC-1) in bronchiolar walls in smokers with and without COPD and non-smokers. Langerin immunoreactivity appears as a diffuse brown color at the cytoplasmic level. **(B)** Number of DC-1 cells/mm^2^ of bronchiolar wall in smokers with severe COPD (blue circles), mild/moderate COPD (orange circles), smokers without COPD (green circles) and non-smokers (yellow circles). Horizontal bars represent median values. Overall comparison by Kruskal-Wallis test (p<0.001) with Dunn’s multiple comparison test. **(C)** Correlation between the number of DC-1 in the bronchiolar walls and the percentage of AM expressing FXIIIA (r=0.43; p=0.018). Severe COPD Blue circles; mild/moderate COPD Orange circles; Smokers without COPD Green circles; Non-smokers Yellow circles.

**Figure 3:**
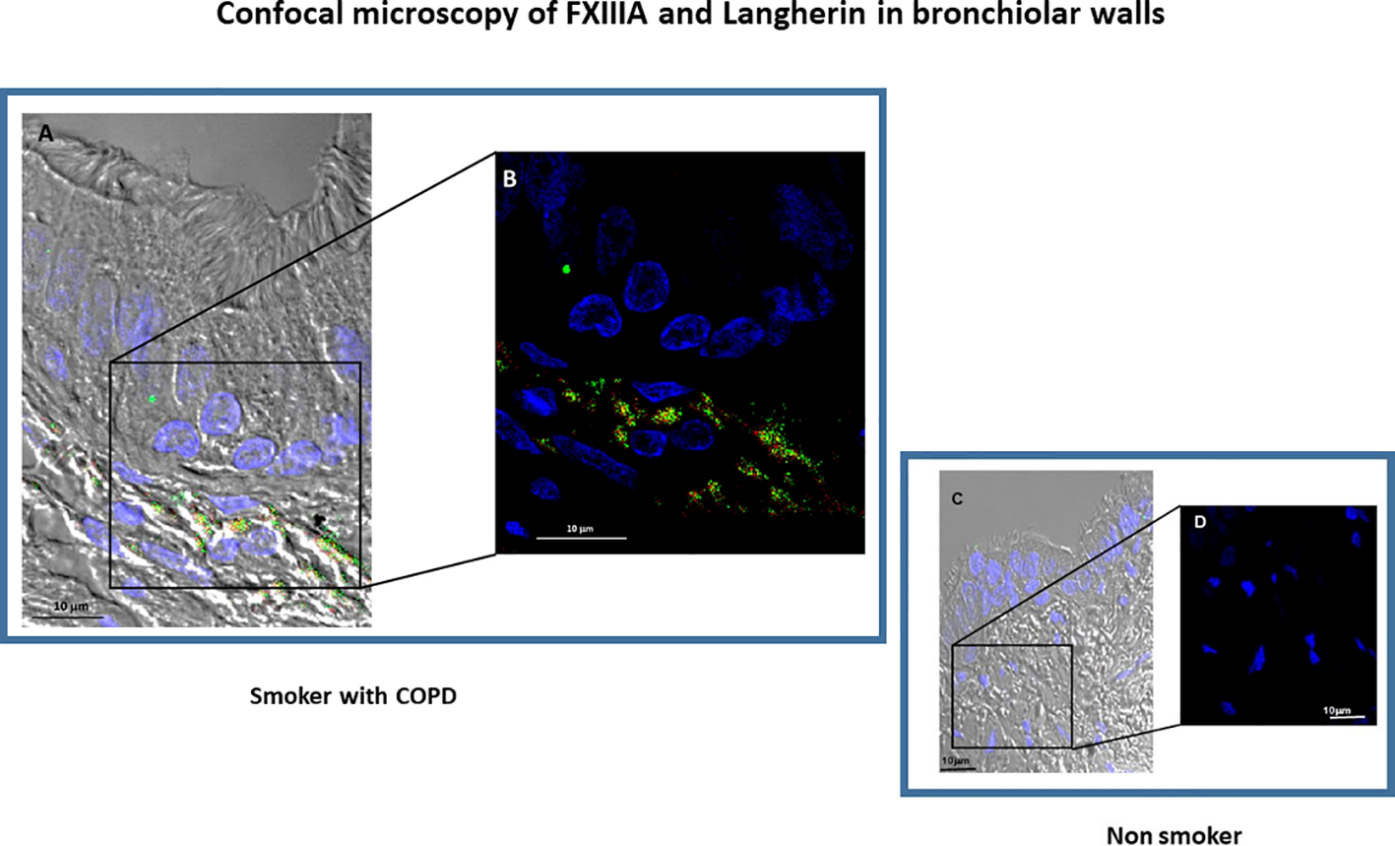
Confocal microscopy of FXIIIA+ and Langherin+cells (dendritic cells, DC-1). Small airway morphology showing epithelium and submucosa by differential interference contrast (DIC) in a COPD subject **(A, B)** and in a non-smoking subject **(C, D)**. The DC-1 cells in the airway submucosa can be identified by the double expression of Langerin (red) and FXIIIA (green). **(B)** shows an enlarged detail from **(A)** confirming the co-expression of FXIIIA (green) and Langerin (red) in the COPD subject, that is not present in the non-smoking subject **(C, D)**. Nuclei were stained with DRAQ5 (blue), FXIIIA (green), Langerin (red). Scale bars= 10 μm.

Since FXIIIA is an important factor for the dendritic cells (DC) development and function (8, 17) we examined the presence of DC in the lung and their relation to FXIIIA and disease severity. Langerin positivity identified DC-1 in the small airways (**Figure 2A**). The number of DC-1 in the small airways was significantly higher in COPD [median(range) 40(0-169) cells/mm^2^] than in smokers without COPD [0(0-29) cells/mm^2^; p=0.05; **Figure 2B**] and non-smokers [4(0-39) cells/mm^2^; p=0.001; **Figure 2B**]. Within the COPD group, DC-1 numbers were significantly higher in severe than in mild/moderate COPD [median(range) 50(36-169) vs 18(0-73) cells/mm^2^; p=0.002 (**Figure 2B**)]. The number of DC-1 in the airways was positively correlated to the percentage of AM expressing FXIIIA (r=0.43; p=0.018; **Figure 2C**). By confocal microscopy we showed that FXIIIA is abundantly expressed in DC-1 in COPD (**Figures 3A, B**), but not in non-smokers (**Figures 3C, D**), a finding not reported previously.

#### 3.2.3 Inflammatory infiltrate in the lung: CD8+ T cells and CXCR3

To assess if the % of FXIIIA+ AM and the numbers of DC-1 were associated with an inflammatory response, we quantified the CD8+ T-cells and CXCR3+cells in the lung.

**Figure 4:**
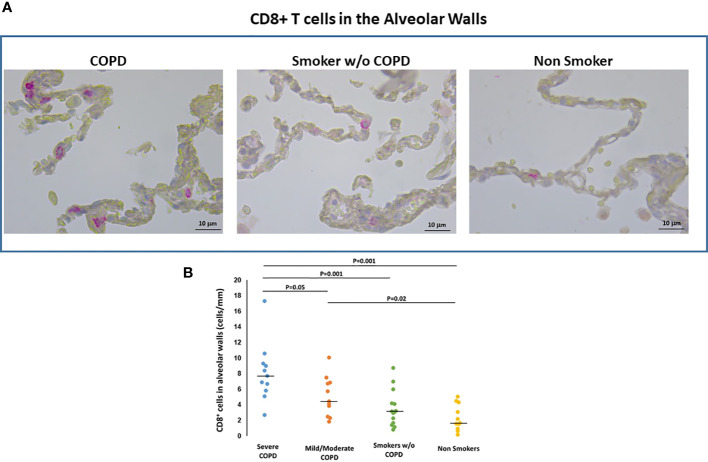
CD8+T cells in the alveolar walls. **(A)** Immunohistochemistry of CD8+ cells in the alveolar walls in smokers with and without COPD and non-smokers. CD8 immunoreactivity appears as a diffuse red color at the cytoplasmic level. **(B)** Number of CD8+ cells/mm of alveolar wall in smokers with severe COPD (blue circles), mild/moderate COPD (orange circles), smokers without COPD (green circles) and non-smokers (yellow circles). Horizontal bars represent median values. Overall comparison by Kruskal-Wallis test (p<0.001) with Dunn’s multiple comparison test.

*CD8+ T cells*. In the alveolar walls, the number of CD8+T-cells was higher in smokers with COPD [median(range) 6.7(2.0-17.5) cells/mm] than in those without COPD [3.2(0.8-8.8) cells/mm; p=0.006] and non-smokers [1.7(0.2-5.1) cells/mm; p<0.001]. CD8+ Tcells were also higher in severe [median(range) 7.7(2.8-17.5) T cells/mm] than in mild/moderate COPD [4.3(2.0-10) cells/mm; p=0.05; **Figures 4A, B**]. There was no difference in CD8+T cells between smokers without COPD and non-smokers (**Figure 4B**).

In small airways the number of CD8+T-cells was higher in severe COPD [median(range) 450(150-558) cells/mm^2^] than in mild/moderate COPD [178(13-389) cells/mm^2^; p=0.02], smokers without COPD [179(72-378) cells/mm^2^; p=0.01] and non-smokers [median(range) 90(25-265) cells/mm^2^; p=0.002].

CD8+ cells in the alveolar walls were correlated with the number of DC-1 in peripheral airways (r=0.61, p<0.001) and with the %FXIII+AM (r=0.49, p<0.001). In peripheral airways, the number of CD8+ cells was correlated with the number of DC-1 (r=0.49, p=0.01).

**Figure 5:**
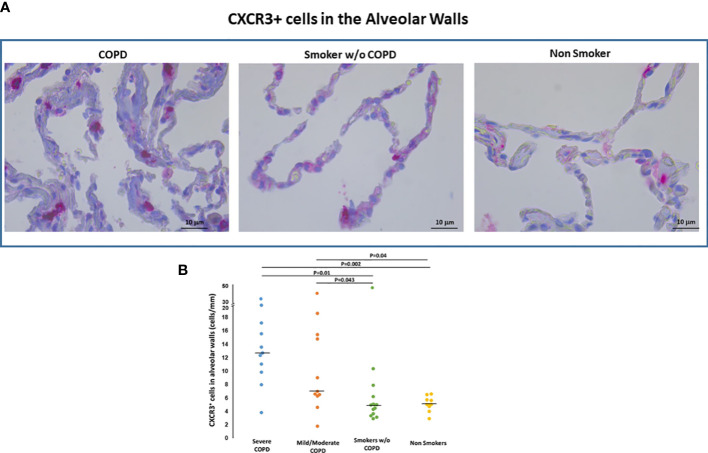
CXCR3+ cells in the alveolar walls. **(A)** Immunohistochemistry of CXCR3+ cells/mm of alveolar wall in smokers with and without COPD and non-smokers. CXCR3 immunoreactivity appears as a diffuse red color at the cytoplasmic level. **(B)** Number of CXCR3+ cells/mm of alveolar wall in smokers with severe COPD (blue circles), mild/moderate COPD (orange circles), smokers without COPD (green circles) and non-smokers (yellow circles). Horizontal bars represent median values. Overall comparison by Kruskal-Wallis test (p=0.001) with Dunn’s multiple comparison test.

*CXCR3+ cells*. CXCR3 immunoreactivity, a marker of activation of the inflammatory infiltrate, was present in AM within the alveolar spaces, in cells within alveolar walls (**Figure 5A**) and in small airways, as previously described by our group (16).

CXCR3+ T cells both in alveolar walls (**Figures 5A, B**) and small airways were higher in COPD [median(range) 11(2-33) cells/mm, 586(145-1320) cells/mm^2^, respectively] than in smokers without COPD [median(range) 5(3-10) cells/mm and 356(176-963) cells/mm^2^; p<0.05, respectively] and non-smokers [median(range) 5(3-6.8) cells/mm, 374(280-615) cells/mm^2^; p<0.05, respectively] as it was the CXCR3 expression in AM [median(range) 73(1-96)% vs 39(9-77)% vs 29(4-80)% p<0.05]. The number of CXCR3+ cells/mm of alveolar wall was positively correlated with the number of CD8+cells both in alveolar walls (r=0.48; p=0.003) and bronchiolar walls (r=0.33; p=0.05).

The number of CXCR3+ cells/mm of alveolar wall was positively correlated with the %FXIII+AM (r=0.32; p=0.03) and with number of DC-1 in small airways (r=0.44; p=0.02).

### 3.3 Morphology/function correlations

**Figure 6:**
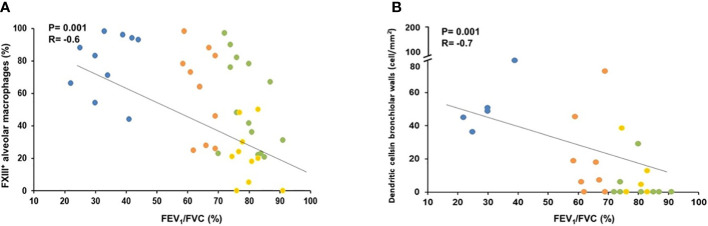
Correlation of lung function (FEV_1_/FVC%) with % of FXIII+ alveolar macrophages and number of dendritic cells in bronchiolar wall. **(A)** FEV_1_/FVC (%) correlated negatively with the percentage of FXIIIA+ alveolar macrophages. Spearman rank correlation (r= −0.6 and p=0.001). **(B)** FEV_1_/FVC (%) correlated negatively with the numbers of dendritic cells in bronchiolar. Spearman rank correlation (r=−0.7 and p=0.001). Smokers with severe COPD (blue circles), mild/moderate COPD (orange circles), smokers without COPD (green circles) and non-smokers (yellow circles).

In order to investigate the possible relevance of the described morphological findings, we correlated them with the degree of functional abnormalities characteristic of COPD -mainly airflow obstruction as measured by the Forced Expiratory Volume in 1 second (FEV_1_) and the ratio of FEV_1_/FVC (Forced Vital Capacity). The significance of the described morphometric findings is highlighted by the correlations found with the pulmonary function abnormalities. Importantly both the % of FXIII+AM (r=-0.6; p=0.001) and the number of DC-1 in peripheral airways (r=-0.7; p=0.001) correlated with the degree of airway obstruction [FEV_1_/FVC% (**Figures 6A ,B**), and FEV_1_% pred: r=-0.5, p=0.001; r=-0.6, p=0.001 respectively) as did the CD8+ (FEV_1_/FVC% and FEV1% pred: r=-0.5, p=0.001 and r=-0.6; p=0.001 respectively) and CXCR3+ cells (FEV_1_/FVC% and FEV1% pred: r=-0.6, p=0.002 and r=-0.5, p=0.001 respectively).

## 4 Discussion

Virtually all forms of tissue damage, regardless of the inciting source or severity of the injury, are associated with local activation of the coagulation system activity and extravascular fibrin deposits, which are not simply a response to the injury to gain hemostasis, but rather are key functional factors in mediating the resulting inflammatory response (1). Thus, factors that influence fibrin stability such as Factor XIIIA, found in bone marrow cells including macrophages and dendritic cells (9), might be an inflammatory modifier specifically in COPD, where macrophages play an important role in the inflammatory response.

We have found and are reporting for the first time that in patients with COPD there is an important expression of FXIIIA in alveolar macrophages (AM) and Dendritic Cells (DC-1). FXIII expression in AM increases with increasing airflow obstruction and positively relates to the number of dendritic cells (DC-1) in the small airways. Both FXIIIA and DC-1 numbers relate with the numbers and activity of T-cells in the lung and with the degree of airflow obstruction, which suggests a significant role of FXIIIA in the mechanism of lung inflammation in response to smoking.

Since the discovery in 1985 of the cellular form of FXIII in monocytes, it has been realized that FXIII is a multifunctional protein that besides hemostasis plays an important role in a wide variety of physiological and pathological processes (6, 18, 19). While circulating FXIIIA2B2 main function is the formation and stabilization of the fibrin clot, the cellular form-FXIIIA- exerts its action mainly at the extravascular level at the sites of tissue injury with the outmost goal being “wound healing”. Activation of the coagulation with the formation of a fibrin clot, recruitment of inflammatory cells and activation of FXIIIA by fibrin would be the so-called “inflammatory phase” of wound healing, a phase that may remain and worsen as the initial stimuli persists, as it happens in COPD (5).

The expression of FXIIIA in macrophages is dynamic in nature, and can be altered in response to external stimulus, by the stimulation with certain immune modulators (20) and possibly by the phenotype of the activated macrophage. It has been shown in “*in vitro”* experiments that upregulation of FXIIIA is induced preferentially in “alternatively” activated (M2) macrophages (in line with the “wound healing” function) while “classical” activated (M1) macrophages- generated in response to stimulation with immune mediators- tend to exhibit downregulation of FXIIIA (20). In our population of smokers with COPD, a Th1 type of inflammatory disease, alveolar macrophages not only showed FXIIIA expression, but the level of this expression increased along with the degree of airflow obstruction and the degree of lung inflammation, indicating that in the “*in vivo”* scenario the impact of the macrophage phenotype ought to be reconsidered. We have previously shown in COPD lungs that the strict division of AM activation in “classical” (M1) and “alternative” (M2) is not clear cut. While we could demonstrate an exclusively M1 phenotype in circumstances of acute inflammation, as the COPD worsened AMs showed a dual M1/M2 combined phenotype, in which the inflammatory stimuli (M1) and the repair stimuli (M2) were expressed simultaneously (15). This phenomenon is what we are probably seeing in the present population, as clearly shown in the confocal images, by the co-expression of CXCR3, a Th1 chemokine receptor, and FXIIIA (a supposed Th2 derivate) in alveolar macrophages. In line with the proinflammatory role of the FXIIIA are the findings of the gene ontology analysis of F13A1 (gene of FXIIIA) which reveals that its expression levels in macrophages are strongly positively correlated to genes responsible for cell differentiation and migration in an immune setup (CCL17, CCL2, CCL13, SERPINB2 and SLC39A8) (21).

As it has been described before (22, 23) we found an increased number of Langerin+ DC in the small airways of patients with COPD, which continued to increase along with disease severity, compared with those without COPD. Furthermore, DC-1 in smokers with COPD express FXIIIA, an important finding never showed before. Alveolar macrophages FXIIIA, which correlated with DC-1 numbers in the airways, may play an essential role in DC proliferation. Macrophages, via the FXIIIA-dependent generation of monocyte chemotactic factor, enhance the proliferation of peripheral blood monocytes, accelerate their migration and significantly inhibit monocyte apoptosis (24). Dendritic cell precursors (monocytes and blood DC) enter the lung from the blood stream under the influence of chemokines, defensins and heat shock proteins produced by the lung tissue (24). Importantly, the differentiation of monocytes into antigen presenting dendritic cells resulted in elevated FXIIIA expression, as we are showing in COPD (17, 20, 24). FXIIIA is essential for DC function since it regulates important intracellular processes involving cytoskeletal remodeling and importantly cell motility, thus enhancing DCs migration to both tissues and lymphatic structures. It has been shown in *in vitro* experiments (8) that, by inhibiting FXIIIA activity, DC motility is severely impaired while FXIII overexpression promotes cell migration. Thus, along with the different chemokines and their receptors attracting DCs to the lung, like CCL20 expressed in the epithelium and its receptor CCR6 (22) and the migratory signature necessary for migration toward follicular lymphoid (25), FXIIIA provides the necessary and essential migratory ability.

As anticipated, a prominent infiltration with CXCR3+ inflammatory cells comprising CD8+ T cells was seen in lung parenchyma and airways (14, 16, 26). The CXCR3 we found expressed in the cells infiltrating the alveolar septa in COPD, is a receptor of the CXCL9, CXCL10, CXCL11/CXCR3 axis for immune response driven mainly by IFN-γ. This axis works primarily for immune cell migration, differentiation, and activation of Th1 cells and CD8+ cells (16, 27, 28). The significance of FXIIIA as a likely factor in the inflammatory response in COPD is highlighted by its correlation with both the number of DC-1 and CD8+ T cells infiltrating the lung. Furthermore, both the CD8+ infiltrate and the expression of CXCR3 were correlated with the number of DC-1 in the peripheral airways, suggesting the presence of a potential inflammatory axis in which FXIIIA might have an important contribution in the inflammatory response in COPD.

Another finding that highlights the prominent role of FXIIIA in the inflammatory cascade described in our cases, is the significant relations found between the severity of COPD as determined by the pulmonary function (FEV_1_/FVC % and FEV1% pred) and all the measured tissue factors (FXIII, DC, CD8, CXCR3). These relations point to a possible role of the inflammatory path initiated by a coagulation that, in individuals with the predisposing endotypes, could progress into COPD.

A possible limitation or criticism of our findings could be that the presence of lung cancer in most of our patients may have influenced our results. We were careful to examine only areas free of disease and distant from tumor, and moreover, since lung cancer was present even in smoking and nonsmoking control subjects, we believe that our findings are valid. Furthermore, patients with severe COPD undergoing lung volume reduction surgery, who did not have lung cancer but had severe COPD, had higher levels of FXIIIA expression than control subjects with lung cancer.

The results of this study suggest that the participation of AM to both the intravascular and extravascular coagulation/inflammation process is important. First alveolar macrophages produce and release into plasma, possibly by extravascular vesicles, the FXIIIA2 component of the intravascular plasmatic FXIIIA2B2. The FXIIIA2 function in the plasmatic FXIIIA2B2 is controlled by the FXIIIB2 fraction produced in the liver and it is only by the detachment of the B fraction from the molecule that FXIIIA2 becomes functional, ensuring the integrity of fibrin and maintaining coagulation. The other function of the FXIIIA2 fraction, produced by macrophages, and by other myeloid and mesenchymal cells, takes place in extravascular tissue sites where the FXIIIB2 fraction is neither expressed nor present. In response to tissue injury, FXIIIA2 has a “wound healing” function by which stabilizing fibrin can initiate tissue repair and inflammation. Importantly patients with FXIIIA deficiency experience impaired wound healing and abnormal scar formation (29).

In conclusion FXIIIA, an important link between the extravascular coagulation cascade and inflammatory response, is significantly expressed in alveolar macrophages and dendritic cells of smokers with COPD suggesting that it could play an important role in the adaptive inflammatory reaction characteristic of the disease.

## Data availability statement

The original contributions presented in the study are included in the article/supplementary material. Further inquiries can be directed to the corresponding author.

## Ethics statement

The studies involving human participants were reviewed and approved by ethics committee University of Padova reference No.0006045. The patients/participants provided their written informed consent to participate in this study.

## Author contributions

Conception and design: ErB, AC, MGC, and MS; performance of experiments: ErB, AC, CR, and MC; clinical characterization: MT, DB, EF, FP, NB, EB, GT; analysis and interpretation: ErB, FC, PSi, FR, PSp, MGC, and MS; drafting of the manuscript for important intellectual content: ErB, AC, PSp, MGC, and MS. All authors contributed to the article and approved the submitted version.
